# Insufficient duration of insecticidal efficacy of Yahe^®^ insecticide-treated nets in Papua New Guinea

**DOI:** 10.1186/s12936-024-05005-x

**Published:** 2024-06-05

**Authors:** Nakei Bubun, Evodia Anetul, Melanie Koinari, Petrina H. Johnson, Leo S. Makita, Timothy W. Freeman, Leanne J. Robinson, Moses Laman, Stephan Karl

**Affiliations:** 1 Papua New Guinea Institute of Medical Research, Madang, Madang Papua New Guinea; 2 Australian Institute of Tropical Health and Medicine, James Cook University, Smithfield, QLD Australia; 3 Papua New Guinea National Department of Health, National Capital District, Port Moresby, Papua New Guinea; 4 Rotarians Against Malaria Papua New Guinea, National Capital District, Port Moresby, Papua New Guinea; 5 Burnet Institute of Medical Research, Melbourne, VIC Australia

**Keywords:** Insecticide-treated nets, Bed nets, Malaria, Papua New Guinea, Yahe, Bioefficacy, Quality, Product evaluation, Durability

## Abstract

**Background:**

Insecticide-treated nets (ITNs) are the backbone of anti-malarial vector control in Papua New Guinea (PNG). Over recent years the quality and performance of ITNs delivered to PNG decreased, which has likely contributed to the stagnation in the malaria control effort in the country. The present study reports results from the first 24 months of a durability study with the ITN product Yahe LN^®^ in PNG.

**Methods:**

The durability study was conducted in four villages on the northern coast of PNG, in an area with high malaria parasite transmission, following WHO-recommended methodology adapted to the local scenario. A cohort of n = 500 individually identifiable Yahe^®^ ITNs was distributed by the PNG National Malaria Control Programme from October to December 2021. Insecticidal efficacy of the ITNs was tested using cone bioassays with fully pyrethroid susceptible *Anopheles farauti* colony mosquitoes at baseline and at 6 months intervals, alongside evaluation of physical integrity and the proportion of ITNs lost to follow-up. A questionnaire was used to collect information on ITN end user behaviour, such as the frequency of use and washing. The observations from the durability study were augmented with simulated laboratory wash assays.

**Results:**

Gradual uptake and replacement of previous campaign nets by the communities was observed, such that at 6 months 45% of all newly distributed nets were in use in their designated households. Insecticidal efficacy of the Yahe^®^ nets, expressed as the percent 24 h mortality in cone bioassays decreased from 91 to 45% within the first 6 months of distribution, even though > 90% of study nets had never been washed. Insecticidal efficacy decreased further to < 20% after 24 months. ITNs accumulated physical damage (holes) at a rate similar to previous studies, and 35% were classified as ‘too torn’ by proportional hole index after 24 months. ITNs were lost to follow-up such that 61% of cohort nets were still present after 24 months. Laboratory wash assays indicated a rapid reduction in insecticidal performance with each consecutive wash such that average 24 h mortality was below 20% after 10 washes.

**Conclusion:**

Yahe^®^ ITNs are not performing as per label claim in an area with fully pyrethroid susceptible vectors, and should be investigated more comprehensively and in other settings for compliance with currently recommended durability and efficacy thresholds. The mass distribution of low quality ITN products with variable performance is one of the major ongoing challenges for global malaria control in the last decade.

**Supplementary Information:**

The online version contains supplementary material available at 10.1186/s12936-024-05005-x.

## Background

Insecticide-treated nets (ITNs) are the mainstay of malaria prevention in large parts of the malaria endemic world, including Papua New Guinea (PNG) [1]. PNG started mass distributions with ITNs in 2006, and routine 3 yearly distributions to all high-burden areas in the country were fully established by 2009 [1]. PNG relied on a single ITN product, PermaNet 2.0^®^ (Vestergaard) between 2006 and 2019. Due to the inconsistent performance of Permanent 2.0^®^ as described in previous studies [2, 3], the PNG National Malaria Control Programme (NMCP) trialled a range of different products, among them Yahe ^®^ LN (Fujian Yamei Industry & Trade Co., Ltd.; henceforth referred to as ‘Yahe’), which was distributed in a range of PNG provinces in 2021.

Yahe is a polyester net coated with deltamethrin, and with a similar active ingredient content as PermaNet 2.0^®^. Given that no pyrethroid resistance has so far been detected in the PNG *Anopheles* populations [4], which mainly belong to the *Anopheles punculatus* species complex, Yahe was an appropriate choice for the PNG NMCP and the donor at the time. Yahe received a time-limited interim recommendation by the former WHO Pesticides Evaluation Scheme (WHOPES) in 2015 [5], due to concerns surrounding the high degree of variability in insecticide content within and between nets. The WHOPES review panel in 2015 further noted “[…] *that the national authorities and procurement agencies must ensure that the Yahe LN complies with WHO specifications* […]” [5]. The WHOPES recommendation was converted to the current WHO prequalification status in 2018. However, it is not obvious whether the time-limited nature of the original interim recommendation still applies.

Very few peer-reviewed studies have evaluated Yahe ITNs since 2015. A 2021 study published by Clegban and colleagues from Côte d’Ivoire concluded that “[…] *Yahe ITNs fulfilled the WHO-PQ criteria for phase II studies of LLINs* […]”, based on evaluations using cone bioassays and experimental huts [6]. This is a surprising statement for an apparently fully prequalified product, which can be assumed to have undergone all stages of testing prior to being granted a recommendation.

Given the scarcity of data available for Yahe ITNs, and in alignment with the above recommendation for further testing, the PNG NMCP, and the PNG Institute of Medical Research, conducted the present evaluation as part of the first distributions of Yahe ITNs in PNG.

## Methods

### Durability study

Yahe ITNs (deltamethrin-coated, 100 denier, L190 cm x W180 cm x H150 cm; WHO PQ listing: https://extranet.who.int/prequal/vector-control-products/yahe-ln) were first distributed in PNG in March 2021 as part of the rolling programmatic mass distributions. Predelivery inspections conducted by TÜV SÜD (Singapore) on a very small number of Yahe ITN samples (n = 3) received at the time indicated that the total deltamethrin content of the ITNs delivered was 1.07 g/kg, which is only slightly above the lower tolerance limit of 1.05 g/kg (1.4 g/kg ± 25%).

The present durability study was layered into the programmatic distributions in Madang Province in PNG, which took place from 08 October 2021 to 12 December 2021. The durability study was conducted in 4 separate villages, named Bulal, Megiar, Mirap and Wasab, on the northern coast of Madang Province (5.2219° S, 145.7856° E), where average monthly temperature ranges from 23.2 to 29.3 °C, humidity is consistently over 85%, and the annual average rainfall is > 3600 mm. The study villages were selected for reliable access and for being representative of coastal (Megiar and Mirap) and inland (Bulal and Wasab) rural PNG. The villages are about 30 km apart from each other and are characterised by highly heterogenous and geographically diverse vector populations characteristic for coastal and inland PNG [7]. A total of n = 500 nets (Batch Number: F2104B2103B2104) were labelled for attrition monitoring by stitching an identification number to their label, and distributed as part of the national program-led distribution. The median number of cohort nets distributed per household was n = 3 (average 2.7) The cohort of ITNs was followed up at 6, 12, 18 and 24 months to confirm ITN presence in the households they had been distributed to. At the time of follow-up, survey teams conducted a questionnaire with the household heads or their representatives. The questionnaire focused on end user behaviour, and was designed, in part, based on templates published as part of The President’s Malaria Initiative (PMI) durability monitoring guidance, [8], but simplified, augmented with scenario-specific questions, and adapted to an electronic data capture system (EpiCollect, version 5.1.52, University of Oxford).

### Measurement of insecticidal efficacy

A target number of n = 30 ITNs that were not part of the attrition-monitoring cohort of nets but that had been distributed at the same time were collected at each follow up time point and subjected to cone bioassays following WHO guidelines [9], using a pyrethroid-susceptible *Anopheles farauti* (Rabaul strain) mosquito colony, as previously described [2]. Briefly, 5 square pieces (one piece from each 4 sides and the roof) were cut from each whole net and pinned to a bioassay board set up at a 45  angle. Each of these pieces was tested using n = 20 unfed, 2–5 days old mosquitoes distributed across n = 4 WHO bioassay cones (n = 5 mosquitoes per bioassay cone). Mosquitoes remained in the bioassay cones for strictly 3 min and were then transferred into holding cups [10]. One negative control, represented by an untreated polyester net purchased at a local supermarket was included in every test. The main outcome measure of the cone bioassays was 24 h mortality, i.e., the percentage of mosquitoes that were killed by the exposure to the net material within 24 h after the exposure. The outcomes were adjusted using Abbotts formula [11]. Negative control mortality never exceeded 10%. The 60 min knock down [9], was also included as a secondary outcome measure.

### Physical assessment

At each time point, a target number of n = 150 randomly selected ITNs were assessed for their physical condition by systematically counting the number of holes in each net and categorising the hole size [12]. The target number was comprised of a random subset (n = 120) of nets monitored for attrition, and those randomly selected and collected for measurement of insecticidal efficacy (n = 30).

The ‘proportional Hole Index’ (pHI), an area-weighted sum of the hole counts, was calculated as described previously [13], to provide an estimate of relative net damage.

### Statistical models

Generalised linear models were constructed to identify which parameters influenced insecticidal efficacy and pHI significantly. Due to the highly overdispersed nature of the count data, negative binomial count models were used. For the model of insectidical efficacy (predicting the number of dead mosquitoes after 24 h), the number of exposed mosquitoes was used as the offset. Parameters that were explored included study village, follow-up time point, wash frequency, usage frequency and the number of users, as well as (for insecticidal efficacy), mosquito age, humidity and temperature at the time of testing. The final models included only the significant predictors [14].

### Simulated wash tests

Laboratory wash tests were conducted on n = 6 new and unused nets from the same batch as those nets distributed in the durability study, as previously described [3] and according to WHO guidelines, with up to 25 washes [9]. Briefly, n = 7 net samples of 25 cm × 25 cm in size were cut from random positions of each whole net. Net samples were introduced individually into 1 L glass bottles (Duran^®^, Sigma Aldrich) containing 500 mL tap water, with 2 g/L local soap (pH 10.5). The soap was added and fully dissolved just before washing. The bottles were placed into a water bath shaker (Julabo SW22, John Morris Group) set to a temperature of 30 °C and shaken for 10 min at 155 movements per minute [9]. The samples were then rinsed twice for 10 min with tap water using the same shaking conditions, dried at room temperature and stored in a laboratory incubator (Heratherm, IMH60, Thermofisher Scientific) at 30 °C in the dark between washes. In order to account for the potential effect of the local soap, parallel wash assays using only water were also conducted. Even though the regeneration time of Yahe ITNs was previously estimated to 1 day, we allowed 3 days between consecutive washes. This more conservative interval between washes was chosen to ensure for full regeneration of surface deltamethrin levels [15].

### X-ray fluorescence analysis on single layers of netting

Proxy element (bromine) content was measured in triplicate in monolayers of netting used for wash tests and collected for measurement of insecticidal efficacy, using a Vanta^®^ (Olympus, Australia) handheld XRF analyser as previously described [16]. To increase the limit of detection, the instrument was used with a shielded measurement chamber and a silica standard background, also sourced from Olympus, Australia. Bromine was quantified by the built-in software and expressed in parts-per-million (ppm). A triplicate measurement took approximately 45 s.

## Results

### Attrition

**Fig. 1:**
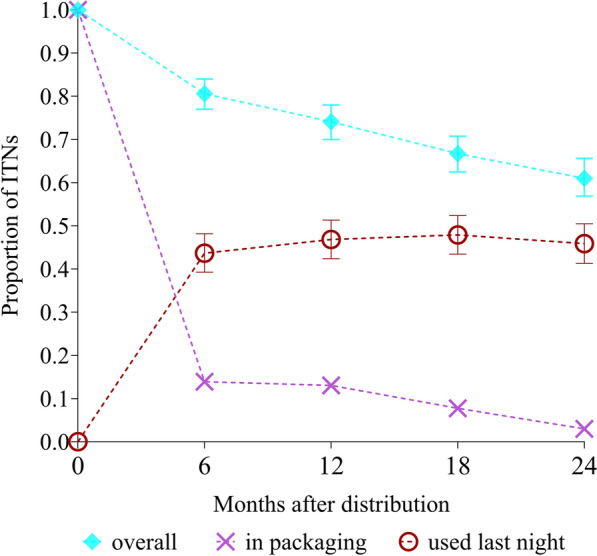
Proportion of ITNs remaining in the community at each follow up time point. The cyan-coloured diamonds represent the proportion of study nets found present at each study time point. The magenta crosses represent the proportion of study nets in packaging that had never been used. The red circles indicate the proportion of study nets used during the night before the survey

The proportion of study nets remaining in their distribution households is shown in Fig. 1. In addition, the figure shows the proportion of study nets that had never been used, and the proportion of study ITNs used the night before the survey.

At 24 months, the proportion of ITNs retained in the distribution households was around 61% (292/476), indicating a ‘median lifespan’ of around 2.5 years [17]. From a total of n = 620 survey responses, the main reasons provided by ITN owners, accounting for > 90% of ITNs lost to follow-up across all survey time points, were ‘given away’ (46.8%, mostly to relatives, 290 responses), ‘used elsewhere’ (17.3%, often in garden houses, 107 responses), ‘never received’ (16.6%, 103 responses) and ‘misplaced’ (10.2%, 63 responses). The authors note that the distribution of all labelled campaign nets was verified directly after the distribution and thus the receipt of all nets by the respective households was confirmed, indicating a self-report bias in favour of the response of ‘never received’. Minor reasons for ITNs lost to follow up included ‘destroyed’ (3.1%, 19 responses), ‘discarded’ (2.7%. 17 responses), ‘stolen’ (2.1%. 13 responses) and ‘alternative use’ (0.6%, 4 responses). Nets that were never used and found to be still in packaging were mostly indicated as ‘reserved for later use’ (> 90%).

### Frequency of ITN washing and ITN washing habits

**Fig. 2:**
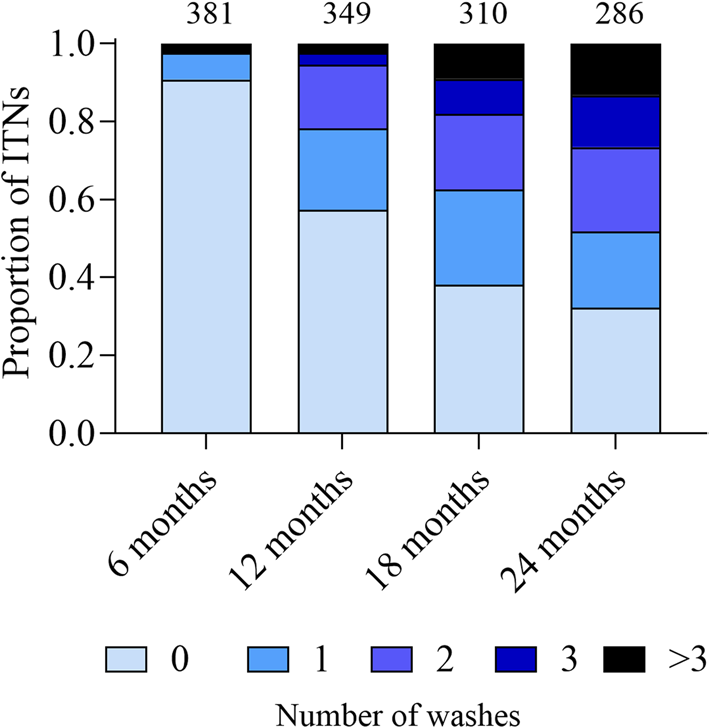
Proportion of ITNs washed at each follow up time point. The bars are stacked. Numbers above each bar indicate the total number of survey responses. The colour scheme indicates the number of washes. Due to the very small proportion of ITNs washed more than 3 times, the category ‘ > 3 washes’ was created

The self-reported frequency of washing of ITNs was low, with 91% (346/381 survey responses) of ITNs reported to never having been washed at 6 months, and 32% of ITNs never having been washed at 24 months (92/286 survey responses). After 24 months, the arithmetic mean number of washes among the ITNs not lost to follow up was 1.8 (range 0 to > 10). Figure 2 shows the proportion of nets washed at each follow-up time point.

The majority of responses in the surveys (85%; 496/664 responses) indicated the use local laundry powder or laundry soap bars to wash ITNs, while 7% (42/664) indicated the use of bleach (often in combination with laundry detergents), and 8% (53/664) indicated the use of only water for washing the ITNs. Over 65% (436/667) of survey responses indicated that drying ITNs in the sun after washing was common, while 8% (56/667) indicated that drying occurred sometimes in the sun, and sometimes in the shade. Only 26% (175/667) indicated to always dry the ITNs in the shade after washing.

### Insecticidal efficacy

**Fig. 3:**
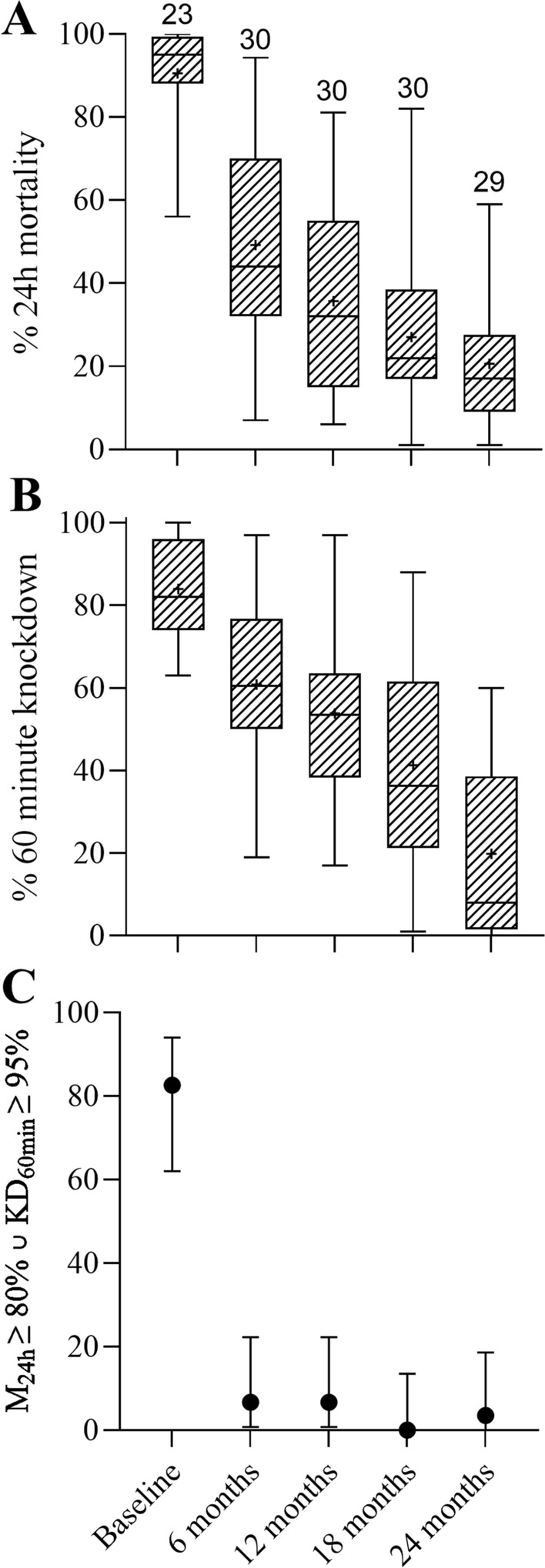
Insecticidal efficacy of Yahe ITNs at baseline and at 6 monthly follow-ups. At each follow-up survey, campaign ITNs were collected from random households in the 4 study villages using a population weighted approach and collecting one ITN per household. **A** Mortality of pyrethroid susceptible *An. farauti* at 24 h after exposure. **B** Knock down of pyrethroid susceptible *An. farauti* colony mosquitoes 60 min after exposure in WHO cone bioassays. The patterned boxes indicate the interquartile ranges, the error bars indicate the ranges, the horizontal lines within the patterned boxes are the medians, while the ‘ + ’ signs are the means. The numbers above the graphs represent the number of ITNs tested. Results from the n = 5 cuttings per ITN were averaged. **C** The proportion of ITNs tested that exhibited ≥ 80% 24 h mortality (M_24h_) or ≥ 95% 60 min knock down (KD_60min_), and the 95% Wald confidence intervals of the proportions

Results of cone bioassays are shown in Fig. 3. Insecticidal efficacy decreased monotonically throughout the study period. At baseline, the average 24 h mortality of Yahe ITNs was 91% (95% CI 86–96%). At 6 months 24 h mortality had decreased to 45% (41–57%), and at 24 months 24 h mortality was just 21% (15–27%). The decrease in insecticidal performance (24 h mortality) could be reasonably well modelled using a one-phase exponential decay function with a half-life of 5.2 months (95% CI 3.5–8.2 months, R^2^ = 0.61). The 60 min knockdown also decreased rapidly. Correspondingly, the proportion of nets with ≥ 80% 24 h mortality or ≥ 95% 60 min knock down decreased from 83% at baseline to 7% after 6 months and 4% at 24 months.

Apart from the follow-up time (i.e., months after distribution), the only significant parameter with influence on 24 h mortality among all nets tested for bioefficacy (n = 124) in the generalized linear model was whether the net had ever been washed. The back-selected model is shown in Table 1.

**Table 1:** Generalised linear model to predict 24 h mortality

Parameter	Exp(B) (95% CI)	p
Never washed (ref. washed)	1.32 (1.04–1.68)	0.022
Months after distribution	0.96 (0.94–0.98)	< 0.001
Intercept	52.09 (38.53–70.44)	< 0.001

While village, frequency of use, and the number of users did not significantly affect insecticidal efficacy and were thus removed from the model, the number of months after distribution and whether the ITN had ever been washed or not had a significant effect

**Fig. 4:**
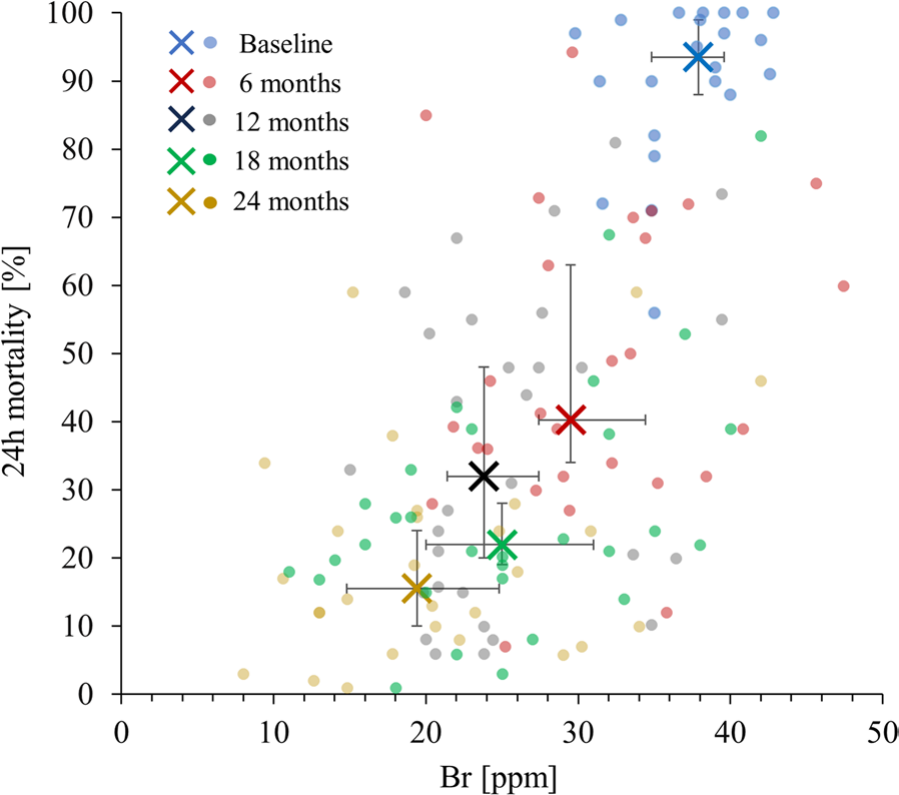
Association between bromine content as a proxy for deltamethrin and 24 h mortality in the Yahe ITNs collected at the follow-up time points. The large ‘X’ shaped symbols and error bars are the medians and their 95% confidence intervals for both bromine content and 24 h mortality at each time point specified by the colour of the symbols. The smaller, circular symbols are the individual measurements for each net sample, with the colour denoting the time point. The Spearman’s rank correlation coefficient is 0.58, p < 0.0001

The insecticidal efficacy of Yahe nets was highly significantly associated with the XRF measurements of bromine, the proxy element for deltamethrin measurable using this technique (p < 0.0001; R^2^ = 0.39) in the samples collected at the follow-up time points. However, the scatter of the data was considerable, and the coefficient of determination was only moderate, indicating that the rapid decay in insecticidal efficacy could only be partially described by this semi-quantitative measurement of insecticide content. Figure 4 shows the association between 24 h mortality and the bromine content in parts per million.

**Table 2:** Generalised linear model to predict the proportional Hole Index

Parameter	Exp(B) (95% CI)	p
Months after distribution	1.12 (1.09–1.16)	< 0.0001
Number of users	1.43 (1.23–1.66)	< 0.0001
Wash frequency^a^	1.19 (1.07–1.31)	0.001
Village (ref.: Wasab)		
Bulal	1.33 (0.85–2.08)	0.212
Megiar	0.61 (0.39–0.98)	0.039
Mirap	1.69 (1.15–2.49)	0.008
(Intercept)	20.26 (7.53–54.55)	< 0.0001

The month after distribution, site (village), number of users and wash frequency were significantly associated with the accumulation of holes

^a^: This model was run separately only for nets that had been washed at least once

### Physical durability

**Fig. 5:**
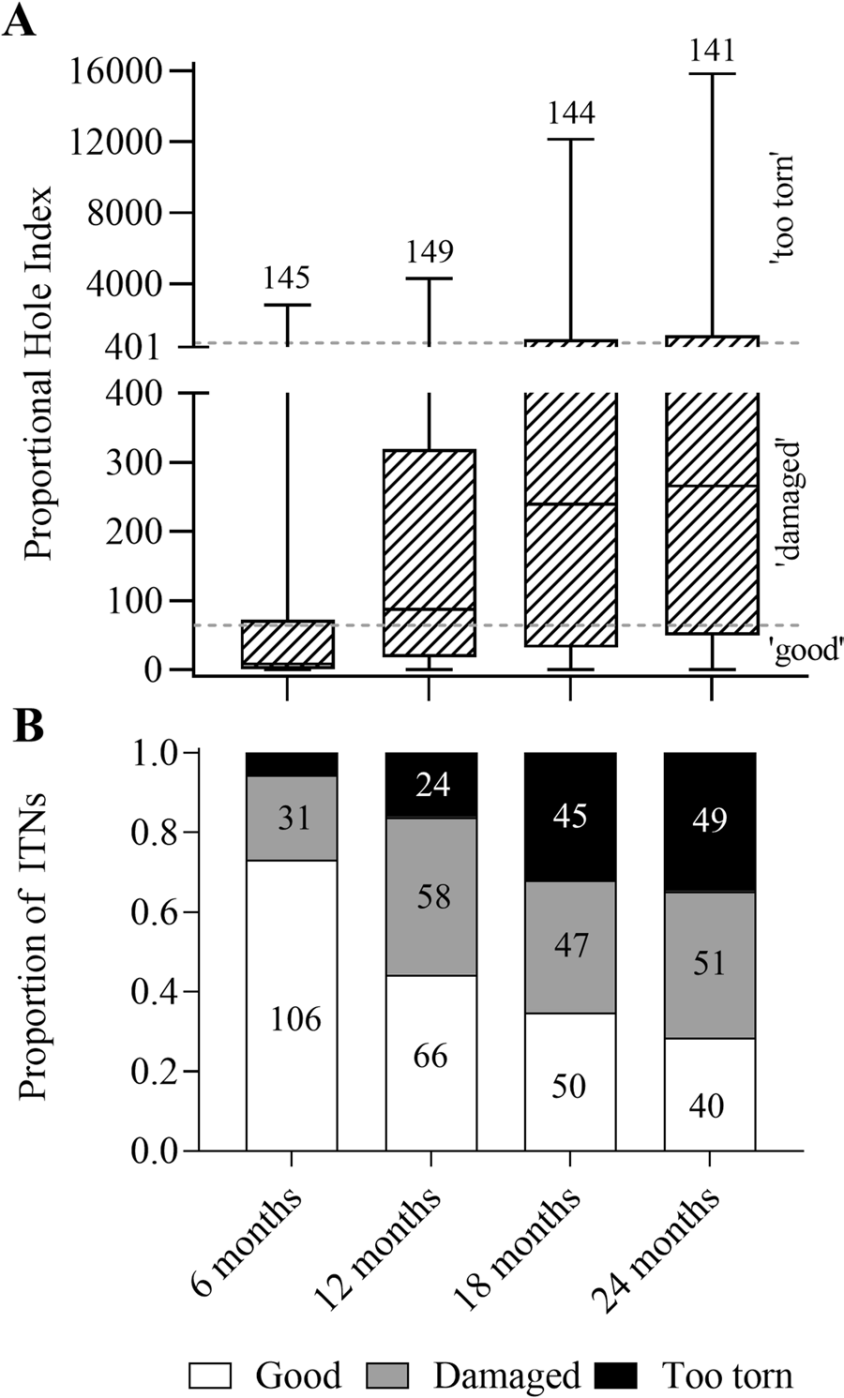
Proportional hole index (pHI) and damage categories of Yahe LN with duration of usage. **A** The pHI was calculated as the estimated area occupied by holes using WHO guidelines [37]. The y- axis was split into two segments with different scales, to allow for better representation of the entire spread of the data. The patterned boxes indicate the interquartile ranges, the error bars indicate the ranges, the horizontal lines within the patterned boxes are the medians. The numbers above the graphs represent the number of ITNs tested. The dashed lines delineate the damage categories of ‘good’, ‘damaged’ and ‘too torn’. **B** Proportion of ITNs falling into each of the 3 damage categories of ‘good’ (pHI < 64), ‘damaged’ (64 ≤ pHI ≤ 642) and ‘too torn’ (pHI > 642). Bar graphs for each time point are stacked. The numbers inside the bars represent the number of nets in each category

The ITNs gradually accumulated damage over time. The proportional Hole Index (pHI; i.e., a measure for the estimated total area of all holes combined in an ITN) is shown in Fig. 4A. Using a previous consensus [12] by which pHI < 64 (equivalent to a total hole area of < 10 cm^2^) is being considered “good condition”,64 ≤ pHI ≤ 642 (total hole area between 10 and 1000 cm^2^) is being considered “damaged condition”, and pHI > 642 (total hole area > 1000 cm^2^) is being considered “too torn to protect from mosquito bites”, the proportion of ITNs falling into these 3 categories at each follow-up time point is shown in Fig. 5 B. After 24 months, 28% of nets (40/141) were in good condition, while 35% (49/141) were in the ‘too torn’ category’.

The statistical model (Table 2) indicated that, in addition to the follow-up time point, the village and the number of users were significant predictors of pHI, i.e., if nets were used by many people, they accumulated holes faster. When only nets that had ever been washed were considered in the statistical model, the frequency of washing was also indicated to contribute significantly to the accumulation of holes. Thus, predictors indicative for a higher usage or handling of nets were significantly associated with the accumulation of holes in the nets.

### Wash resistance of insecticidal efficacy

Insecticidal efficacy expressed as 24 h mortality of pyrethroid susceptible *An. farauti* mosquitoes in standard cone bioassays decreased exponentially with consecutive washes, as shown in Fig. 5A, with a half-life of 2–3 washes (model estimate 2.7). Insecticidal efficacy reached < 20% 24 h mortality after around 10 washes. The spread of the data was considerable, with two of the six nets still exhibiting ≥ 50% 24 h mortality after 25 washes.

**Fig. 6:**
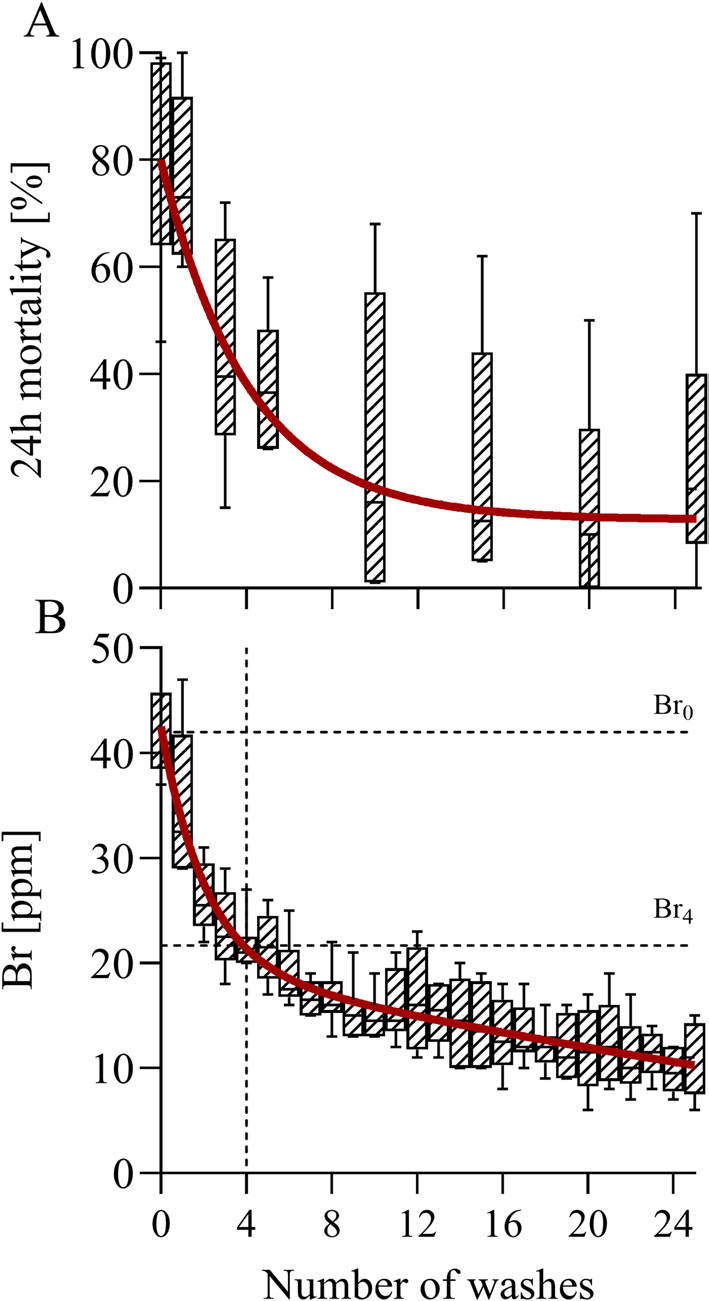
Wash resistance of insecticidal efficacy and proxy for wash resistance index using XRF. **A** 24 h mortality after washing of Yahe ITNs. A total of n = 6 Yahe ITNs underwent 25 standard washes with a local soap (pH = 10.5). The red line is a biphasic exponential decay function to serve as a guide to the eye (the actual fit is poor). **B** Bromine quantification in the same ITNs by XRF, correlated to the number of washes. The wash resistance index is the average loss of insecticide per wash over the first 4 washes, indicated by the dashed lines in **B** The red line is a biphasic exponential decay function (R^2^ = 0.82)

The XRF measurements of bromine as a proxy for deltamethrin indicated a monotonic decrease of insecticide in the ITN samples with each consecutive wash (Fig. 5B), which could be described well with a two-phased exponential decay function (R^2^ = 0.82). These measurements were also used to calculate an estimate for the wash resistance index [15] (Fig. 6). The wash resistance index estimate based on the model fit to the XRF data was 86%.

## Discussion

The present study evaluated the durability of Yahe^®^ ITNs distributed in late 2021 in Papua New Guinea (PNG). This is the first longitudinal ITN durability study conducted in PNG. Over 1,401,000 Yahe ITNs were distributed in the country to protect > 2.8 million people from malaria for 3 years.

With > 60% of study nets still present at 24 months after distribution, and a median half-life of around 2.5 years, the data indicate that ITN retention in the 4 survey communities was higher than observed in previous studies, conducted with various products and in various settings summarised in a recent review, which found that > 75% of previous durability monitoring studies reported median retention times of < 2.4 years [18]. The present study shows that around 34% of ITNs had either never been utilised by 6 months (14%) or were already lost to follow-up (20%) after 6 months. However, of the nets lost to follow-up > 60% were indicated to have been given away to other users (most were given to family), or used as intended elsewhere (e.g., in garden houses), which indicates that these ITNs were likely not lost to the overall control effort. Only a very small proportion of nets were reported to have been destroyed, stolen or used for other purposes, but there was a reporter bias occluding the reason for around 16% of ITNs lost to follow up. These ITNs were reported as ‘never received’, yet receipt had been confirmed by the study teams.

The present study indicates that ITNs were washed relatively infrequently in the study sites in PNG, as compared to other studies [19–21], with over 90% of ITNs never having been washed in the first 6 months and a median number of washes of 1 (IQR 1–3) at 24 months after distribution. Most people used laundry powder or laundry soap bars to wash the ITNs but only a small percentage (7–8%) resorted to adding bleach. The majority of responses (> 65%) indicated drying of ITNs in the sun, and it appears that including more information around the need to minimize exposure of ITNs to sunlight into behavioural change communication may be beneficial [22].

With only < 10% of the ITNs experiencing any washes within the first 6 months after distribution, it is all the more surprising that the insecticidal efficacy, as measured in cone bioassays with fully susceptible *An. farauti* colony mosquitoes decreased rapidly to less than 50% within this period (Fig. 3). This indicates that the Yahe ITNs distributed in PNG were inherently unable to retain their insecticidal efficacy under the conditions prevailing in these PNG sites, which are alike to those in many tropical settings where ITNs are used, with average temperatures ranging from 23.3 to 29.3 °C and humidity > 85%. The strong correlation between bromine as a proxy for deltamethrin, and 24 h mortality in the net samples collected during follow-up (Fig. 4) indicates that at least some of the observed rapid decay of insecticidal efficacy is due to the loss of insecticide. However, it needs to be noted that bromine content is not equivalent to deltamethrin surface concentration as the XRF is only a semi-quantitative measurement of total AI content (not surface AI content), and because a partly destroyed or even just isomerized deltamethrin will still contribute to the bromine reading by XRF, even though has no insecticidal effect.

Given most of the nets had been washed less than 3 times at the end of the 24 months period, the mechanism of this insecticide loss remains elusive. It would be important to better understand this mechanism as it appears that simply washing ITNs is not enough to simulate this form of ITN decay or aging. Killian and colleagues noted in 2008, that insecticide loss over time could not fully be explained by washing and attributed the remaining variation to ITN ‘handling’ [23]. While this is plausible and supported by the findings in the present study, further studies are required to fully understand the main causes of insecticide loss in ITN durability studies.

Yahe ITNs accumulated physical damage such that 35% of nets were too torn to protect from mosquito bites at 24 months. While this is context-specific and not easily generalizable, this proportion seems to be within the range of observations made in similar studies on pyrethroid-only nets, [24–28]. The number of net users (i.e., the number of people regularly using the same net) was significantly associated with physical degradation. This correlation emphasises the need to maximise ITN coverage and physical durability [29], and reconsider current assumptions regarding target net per person rates in distribution campaigns [17].

Simulated washing of Yahe ITNs lead to unacceptably rapid decay in insecticidal efficacy of the ITNs in the present study, with a median 24 h mortality of < 20% after 10 washes. These findings were complemented by XRF measurements indicating a rapid decrease in the insecticide concentration over the first few washes, which could be described well by a biphasic exponential decay function.

The XRF measurements indicated that the ITNs had an average wash resistance index of around 86% (the wash resistance index being the average loss of insecticide over the first 4 washes [30]). The current specifications for Yahe ITNs require a wash resistance index of only 85% [31], which, in general, seems unacceptably low as it means that after 20 washes only 3.9% of the original active ingredient concentration is expected to remain (i.e., around 2.2 mg/m^2^), which is far below the minimum effective concentration of 10 mg/m^2^ estimated in previous studies for deltamethrin coated nets [32].

The present study had important limitations. It is conceivable that the need to label the study nets due to the absence of net-specific barcodes on the label of Yahe ITNs may have led to a bias in the attrition rate as study participants may have been more aware of these nets being part of a study. However, there is no evidence from previous durability studies that would suggest that such a bias exists. The present study purposefully did not remove the nets from their original plastic packaging at the time of distribution as this is not done during the programmatic distributions in PNG. This may also have contributed to the relatively long observed median half-life. However, it also enabled a more realistic observation of the gradual uptake of the newly distributed campaign nets in the communities. This may be useful for mathematical modelling studies endeavouring to model the impact of ITN distributions. Models frequently assume that all ITNs are accessible immediately to all potential users at the time of distribution, which may be an unrealistic assumption [33, 34].

While the XRF technique employed in the present study is not equivalent to the methodology developed by the Collaborative International Pesticides Analytical Council (CIPAC) to determine the wash resistance index, it can be considered a very good approximation. In addition, this study did not conduct further chemical analyses such as HPLC to determine changes in the isomeric state of the deltamethrin.

## Conclusion

Overall, the present study indicates that the insecticidal efficacy of Yahe ITNs is very short-lived in PNG settings, and the aspirational 80% 24 h mortality previously recommended by WHOPES as a performance criterion for used ITNs after 3 years is only achieved on average and when the nets are completely new. Thus, while these nets still present a physical barrier between users and mosquitoes when they are not damaged, it is unlikely that they would be able to unfold a sustained community effect by impacting mosquito populations [35, 36].

Importantly, there was no strong indication that end user behaviours such as washing would be responsible for this, as most of the decay occurred in the first 6 months and in ITNs that had never been washed. It rather appears that the product is inherently unable to retain insecticidal efficacy, with data perhaps suggesting that drying in the sun may contribute slightly to the loss of insecticidal efficacy in later years of the study. The possibility that some of the cohort ITNs had a low baseline deltamethrin content cannot be excluded, given the predelivery inspection data provided, which would be equally worrisome. However, in view of the rapid loss of total AI and insecticidal efficacy of the product, it appears unlikely that the label claim *‘Yahe net last for at least 20 washes or 3 years’* (as shown in Supporting Information Fig. 1), as it is stated on the product packaging, is justified.

The product should be further investigated for compliance with present performance requirements. PNG cannot be considered a unique environment, and Yahe nets sent to PNG are unlikely to be different to those delivered to other countries. Other countries and donors therefore should re-evaluate whether Yahe ITNs are suitable to protect target populations against malaria in their settings. Better quality control processes for ITN products are urgently needed.

### Supplementary Information

Supplementary material 1 Figure S1: Packaging of Yahe ITNs recovered from the present study. The label claim ‘Yahe net last for at least 20 washes or 3 years’ is not supported by the results of the present study.

## Data Availability

The datasets used and/or analysed during the current study are available from the corresponding author on reasonable request.
