# Supplementary figures and images for: Insufficient duration of insecticidal efficacy of Yahe® insecticide-treated nets in Papua New Guinea
Source: Malar J. 2024 Jun 5;23:175. doi: 10.1186/s12936-024-05005-x (PMC11151609; doi:10.1186/s12936-024-05005-x)

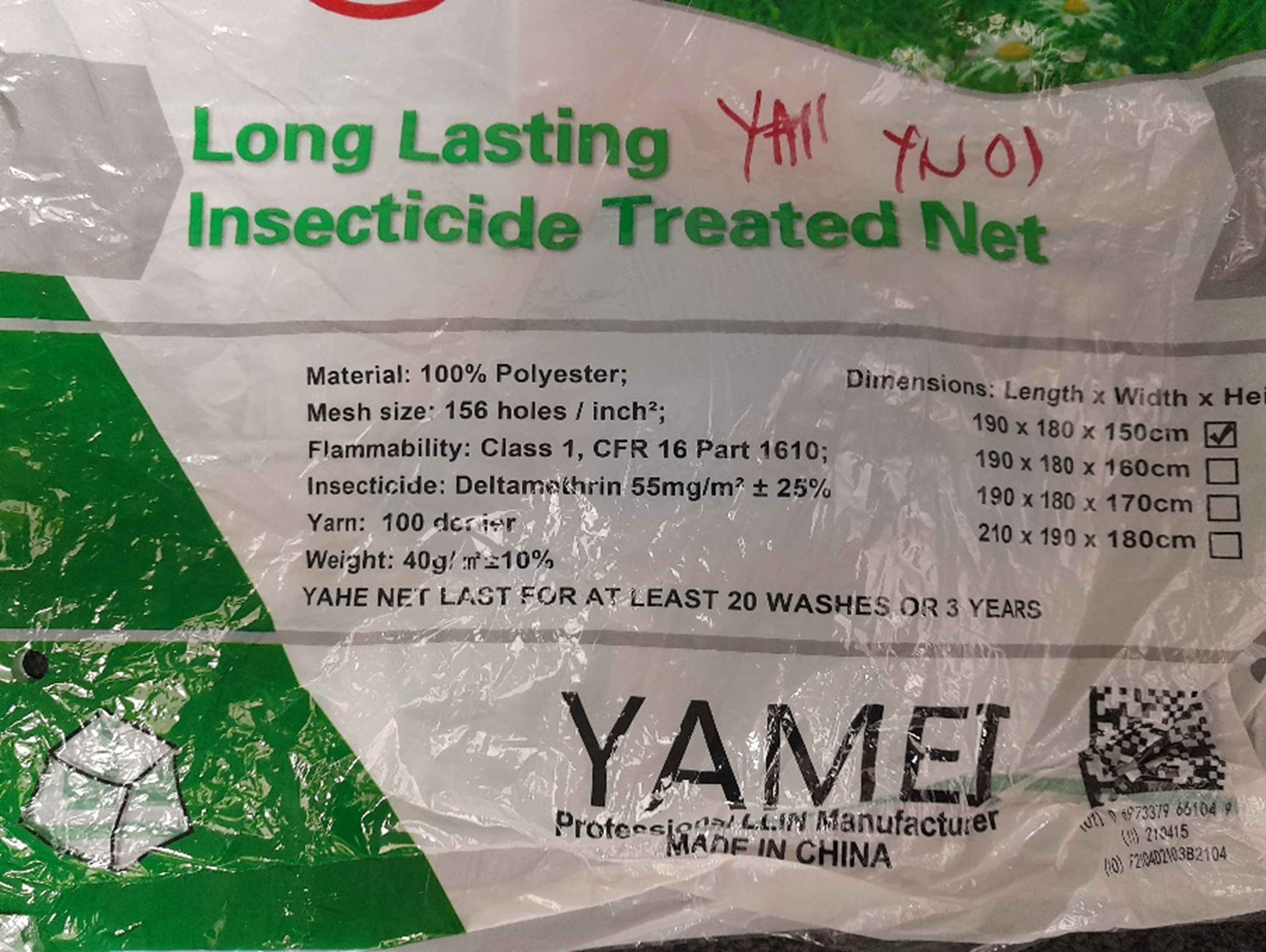

Supplement: Supplementary file 1 — Supplementary material 1 Figure S1: Packaging of Yahe ITNs recovered from the present study. The label claim ‘Yahe net last for at least 20 washes or 3 years’ is not supported by the results of the present study. [file 12936_2024_5005_MOESM1_ESM.tif]
